# Science teacher beliefs in conflict-affected zones of Jammu and Kashmir

**DOI:** 10.1007/s11422-023-10181-4

**Published:** 2023-04-28

**Authors:** Rehka Koul, Garima Bansal

**Affiliations:** 1 School of Education, Curtin University, Kent St, Bentley, WA 6102 Australia; 2 Australian Council for Educational Research, Unit 406 & 407, Southern Park. D-2, District Centre, Saket, New Delhi, 110 017 India

**Keywords:** Conflict-affected zones, Jammu and Kashmir, Teacher beliefs, Science, Fragile contexts

## Abstract

This study examines science teacher beliefs working in the conflict-affected zones of Jammu and Kashmir. Research in these areas indicates that teacher beliefs influence their classroom practices and student learning outcomes and that teacher beliefs are highly context sensitive. Using data collected from a questionnaire and focussed group discussions, this research elucidates science teachers’ beliefs regarding how conflict affects classroom practices, conflict and teaching issues, the multifaceted role of teachers in conflict-affected zones, the role of science education in ameliorating conflict, and the changing role of teachers during three decades of active conflict in Jammu and Kashmir. A nuanced picture of teacher beliefs originated from this study suggesting that despite challenges teachers are committed to children’s academic, cognitive, and psychosocial growth.

## सारांश

विभिन्न शोधों में यह बताया गया है कि शिक्षक की मान्यताएं उनके कक्षा अभ्यासों और अधिगम परिणामों को प्रभावित करती हैं। साथ ही, यह भी बताया गया है कि शिक्षक की मान्यताएँ अत्यधिक संदर्भ-संवेदनशील होती हैं। यह अनुसंधान तीन दशकों से सक्रिय संघर्ष-प्रभावित जम्मू और कश्मीर के क्षेत्रों में काम कर रहे विज्ञानं शिक्षकों की मान्यताएं का अध्ययन करता है। एक प्रश्नावली और केंद्रित समूह चर्चाओं से एकत्र किए गए डेटा का उपयोग करते हुए, यह शोध विज्ञान शिक्षकों की मान्यताएँ को स्पष्ट करता है कि संघर्ष कैसे कक्षा प्रथाओं और शिक्षण मुद्दों को प्रभावित करता है। यह अनुसंधान संघर्ष प्रभावित क्षेत्रों में शिक्षकों की बहुमुखी भूमिका, उनके संघर्ष को कम करने में विज्ञान शिक्षा की भूमिका, और जम्मू और कश्मीर में तीन दशकों के सक्रिय संघर्ष के दौरान शिक्षकों की भूमिका में आए परिवर्तन को दर्शाता है। इस अध्ययन से शिक्षकों की मान्यताओं की एक सूक्ष्म तस्वीर सामने आई है जो यह बताती है कि चुनौतियों के बावजूद शिक्षक बच्चों के शैक्षणिक, संज्ञानात्मक और मनोसामाजिक विकास के लिए प्रतिबद्ध है।

Countries with fragility, conflict, or violent contexts represent the biggest challenges to achieving the United Nations Sustainable Development Goals of ensuring inclusive and equitable quality education and promoting lifelong learning for all (World Bank 2018). Children in these regions are exposed to violent extremism in their early developmental stages. Such experiences affect their attitudes, values, and behaviours for a lifetime.

Historically, the state of Jammu and Kashmir (J and K) now a unionised territory, has suffered extensive political instability since India’s partition and creation of Pakistan in 1947. Shubh Mathur (2016) noted that the state since being claimed by both India and Pakistan has resulted in three major wars and, a state of continuous unrest since 1989 that has resulted in a massive loss of life, the exodus of half a million Kashmiri pandits, horrific abuses against women and children, and a greatly debilitated national infrastructure that has left state institutions weak. Bill K. Koul (2020) observed that conflict has persisted in the state of J and K such that it has ensured protracted insecurity and political instability since 1989. A report by Special Broadcasting Services (2019) mentioned that Kashmir currently is the most militarised zone in the world and terrorist activities are common occurrences in the State. Contextually, this ongoing situation puts a great strain on the education systems which could play an instrumental role in alleviating this militarised setting.

Education can be an important tool to extirpate divisions and tensions along religious, political, social, and ethnic lines by mitigating the risks associated with such adversity and help children and youth to succeed despite severe challenges (World Bank 2018). Toshio Ohsako (1997) while suggesting possible interventions to be made in schools located in violent contexts observed that science education lends itself to developing critical thinking skills among young people, hence, they can distance themselves from extremism and resist the ‘pull factors’ through awareness-raising, generating respect for others, and creating and maintaining cultures of peace and dialogue. Of course, in order to reap the rewards of education, science teachers must include pedagogical activities that foster critical thinking skills among students.

Jackie Kirk (2008) noted that teachers, who are often part of the same community, share similar distorted historical baggage as that of children. All the more, they work in insecure circumstances where sporadic attacks are commonplace. The environment of fear and oppression influences their beliefs about science teaching, students, and other school and community variables. These beliefs have a concomitant effect on classroom practices. Accordingly, this study understands teacher beliefs as a recursive contextual co-construction that takes place daily in educational contexts, situated within the larger socio-cultural-political-economic-historical factors of the society. This study examines teacher beliefs in conflict-affected zones of J and K.

## Jammu and Kashmir: a continuous state of unrest

### Historical background

**Fig. 1:**
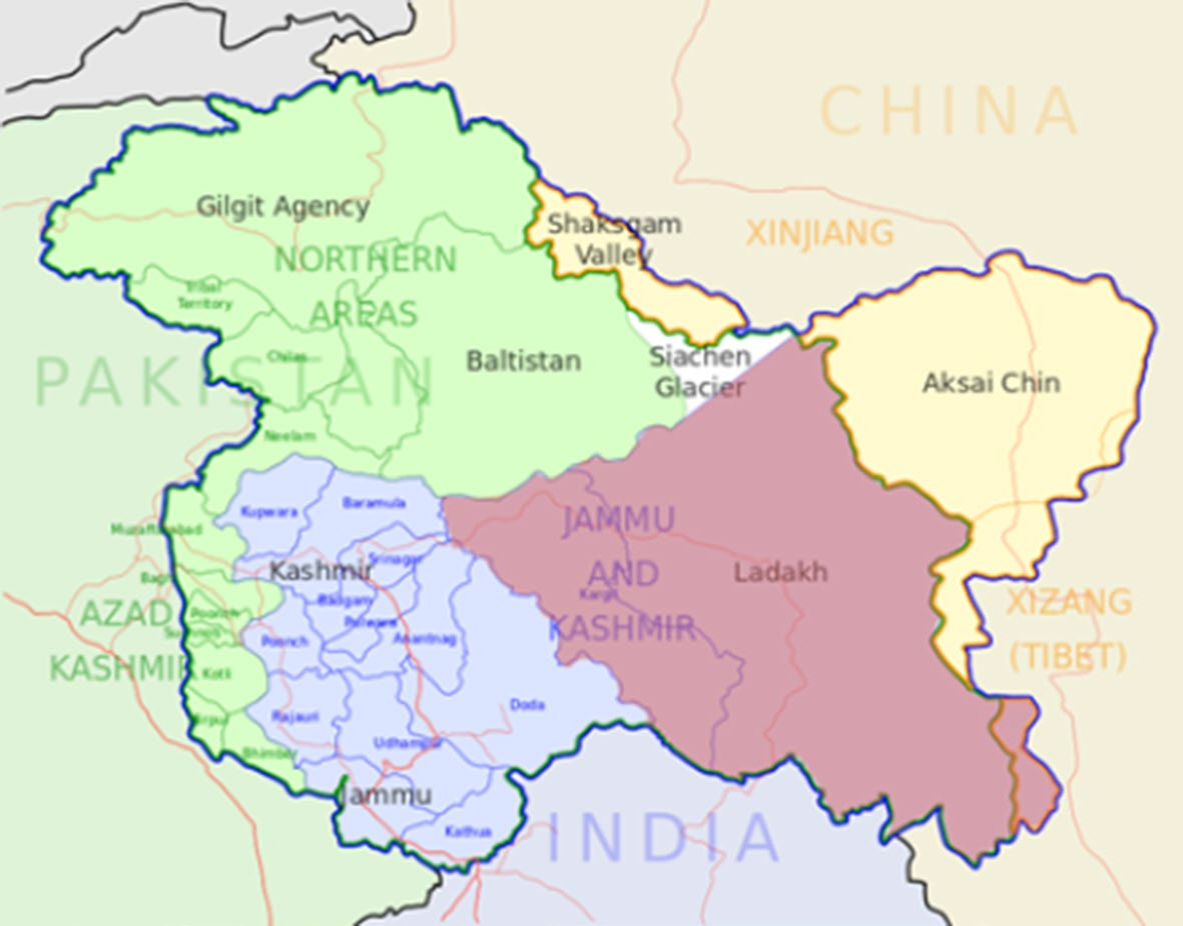
Map of Jammu and Kashmir

The history of the state of J and K is interwoven with the history of the wider Indian subcontinent and its surrounding areas, including Central Asia, South Asia, and East Asia (Fig. 1). The state has all throughout maintained a very complex history in terms of religions, cultures, and rulers. For example, in the first half of the first millennium, Kashmir became an important centre of Buddhism and later Hinduism. In the late ninth century, a particular Hindu sect, Shiva, rose and Buddhism declined in the state. Islam was introduced to Kashmir by Sufi saints between the 13th to fifteenth centuries and over time became the most practised religion. In 1339, Shamir became the first Muslim ruler of Kashmir and opened the Shamir dynasty. For the next five centuries, Muslim monarchs ruled Kashmir, including the Mughal Empire, which ruled from 1586 to 1751, and the Durrani Empire of Afghanistan, which ruled from 1747 to 1819. However, today all the religious groups coexist in the state.

The J and K gained statehood in 1846, after the Sikhs were defeated in the first Anglo-Sikh War and they signed the Lahore Treaty, wherein Raja Gulab Singh purchased the area from the British and became the new ruler of the region. His descendants ruled under the supremacy, or guardianship, of the British royal family until 1947. The state comprised three distinct areas of Jammu, Kashmir, and Ladakh. All three regions are diverse in terms of geography, religion, culture, and language (see Table 1).

**Table 1:** Diversity in the state of Jammu and Kashmir

Region	Topography	Religion	Language	Costume	Food/Staple
Jammu	Sub-tropical	Hindu	Dogri	Chudidar/Kurta	Roti
Kashmir	Alpine	Muslim	Kashmiri	Phiran	Rice
Ladakh	Cold and Dry	Buddhist	Ladakhi	Guncha	Noodles

### Merging with India

In August 1947, the subcontinent was partitioned into two countries, India and Pakistan, based on religious demographics. However, the king of Kashmir, Maharaja Hari Singh, a Hindu by religion while governing a Muslim majority state was indecisive and did not join either country. In October 1947, Pakistani militia raided the state and Maharaja Hari Singh signed accession with India in lieu of India providing defence to the state of J and K. Accession to India was signed giving Kashmir a special status of greater autonomy and was supported by the popular leader Sheikh Mohammad Abdullah who later governed the state as Chief Minister. This special status was given under Article 370 of India’s Constitution conferring J and K with the power to have a separate constitution, a state flag and autonomy over the internal administration of the state. Currently, part of Kashmir popularly known as Azad Kashmir and Gilgit-Bal has ended up under the jurisdiction of Pakistan territory and part of Ladakh Aksai Chin and Trans-Karakoram region is under the jurisdiction of China (see Fig. 1) (Koul 2020). Since 1947, India and Pakistan have fought three wars over Kashmir, and the tension continues. This continuous conflict makes the Indo-Pak border zone the world’s highest militarised area, and the allegiances of the local population is divided which complicates establishing a peaceful atmosphere (Koul 2020).

### Times of raging conflict

Following a gradual collapse of unmet expectations of the Kashmiri population, an armed militancy supported by Pakistan grew in India-administered-Kashmir towards the end of 1980’s. As described by Koul (2018) on the 19th of January 1990, all non-Muslims living in Kashmir were directed by militant organisations in an organised manner to leave Kashmir, resulting in nearly half a million people leaving Kashmir for the nearest region Jammu in a short period.

In reaction to the armed conflict and mass exodus of the Hindu community from the Kashmir region, India dissolved the local government in late January 1990 and imposed a centrally enforced government. Politically things have changed since 1990 and intermittently the region has been governed by elected government representatives. However, a desire to either join Pakistan or achieve total freedom from India continues to storm the mindsets of certain sections of the Kashmiri diaspora. These sections of society spurred militancy in the region. And, to avoid further conflict in the region, the Indian government is forced to maintain armed forces in the region.

### Abrogation of Article 370

On 5 August 2019, the Government of India abrogated Article 370, bifurcating the state in two parts (1) Jammu and Kashmir and (2) Ladakh. This was followed by taking away the statehood and making them Union Territories under the auspices of the Government of India.

Pre abrogation of Article 370, except for defence, communication, foreign affairs, and finance, each state leader had power over their state without the impending influence of the Indian central government. The assembly of the state made its laws for land and properties, and people from outside of the state were banned from buying land in the state.

Now that the special powers associated with Article 370 are taken away, the whole region is under the control of the Indian central government. The Armed Forces Special Powers Act (AFSPA), which gives powers to the Indian army to detain anyone suspicious without any kind of accountability and trial, is put in place in certain sensitive regions of the territory. Abrogation of Article 370 has caused enormous amounts of unrest in the state with curfews being imposed for nearly a year, earning the mistrust of people.

## Education in Jammu and Kashmir

In ancient Kashmir, local priests ran educational institutions, and science was one of the major disciplines of instruction. During the British colonisation, the present system of education came into existence and at present, this is the only officially recognised educational system. Prem Nath Bazaz (2011) noted that the first school in Kashmir was started by J. H Knowles in 1881, which can be termed a red letter day in the history of modern Kashmir. A decade later a girl’s school was started, which posed its own challenges by the then conservative society. In the first decade of the twentieth century Mrs Annie Besant, then president of the Theosophical Society, laid the foundation for a higher education college, which was subsequently taken over by the state authorities (Beg 2021).

In the last century, there has been a gradual increase in the number of educational institutions in the state of J and K that has resulted in a literacy rate of 67.16% (GoE 2011). The emphasis has been on increasing and improving the education system to create growth opportunities for the local people, thus raising the economy (Aditi 2020). The state has more than 1000 schools for primary, secondary, and senior secondary education. In addition, more than 50° giving colleges and 12 universities serve the higher education requirements. The government and private colleges of the state offer degrees in science, arts, and commerce streams.

## Review of science teacher beliefs

Lynn A. Bryan’s (2012) research over the years has observed that despite innovative education policies being launched by education ministries across the world, hardly any purposeful instructional change can be observed in the classrooms. One of the prime reasons for poor implementation of innovations in classrooms, as observed by the research conducted on teacher beliefs by Andrew T. Lumpe, Jodey J. Haney, and Charlene M. Czerniak (2020) is the implicit resistance offered by teacher beliefs. Rodger W. Bybee (1993) cautioned the science education community to focus on teacher beliefs if they want to see any reform in the science classroom practices.

Teacher beliefs have been defined differently across the literature. They are often equated with knowledge, attitudes, personal convictions, perceptions, experiences, or reflect a person’s acceptance or rejection of a proposition (Bryan 2012). M. Frank Pajares (1992) observes that beliefs are the stepping stones to human behaviour. He explained that clusters of beliefs around a particular situation form attitudes, and attitudes become actions that guide persons’ decisions. This study understands teacher beliefs as a recursive contextual co-construction that takes place every day in educational contexts, situated within the larger socio-cultural-political-economic-historical factors of the society.

Science teachers’ beliefs about their science teaching are constituted by their self-efficacy to teach science, attitudes and interest towards science, and contextual factors including student variable, work environment, community variables, and other national and global variables (Lumpe, Haney, and Czerniak 2000). Bryan (2012) pointed out that the context of the teacher includes how the teacher perceives his/her world as well as the teaching conditions that teachers must negotiate daily. To this, Albert Bandura (1993) adds that the whole school environment influences teacher’s beliefs since teachers do not operate in isolation. Numerous studies, including Garima Bansal, Umesh Ramanarain, and David Schuster (2019) documented how other external conditions, for example, strict accountability, a culture of time efficiency, mandatory curricula, state and national assessments, teacher socialisation, etc. influence teacher beliefs, eventually affecting their classroom practices.

## Science teacher beliefs in conflict-affected zones

Reviewing the related literature on science teacher beliefs in conflict-affected zones, it emerged that a limited amount of the literature surrounding teacher beliefs in fragile settings exists. Therefore, it is necessary to adopt a broader approach. Literature on related constructs, such as teacher motivation and demotivation, teacher stress, teacher experiences, and teacher well-being in fragile settings, is also drawn upon for possible insight into teacher beliefs in conflict-affected zones.

Rebecca Winthrop and Jackie Kirk (2005) observed that teachers teaching in conflict-affected areas have a critical role to play in children’s physical, cognitive, and psychosocial development. To this, Susan Nicolai and Carl Triplehorn (2003) added that teachers working in conflict zones are endowed with the responsibility of creating a safe learning space where children are free to share their concerns with their peers and trusted adults with opportunities to be creative and healthy in difficult environments. However, Y. Abisola Noah-Pinheiro’s (2017) observed that the teachers teaching in these areas often themselves face low levels of motivation and high levels of frustration and uncertainty. INEE’s (2010) report mentioned that teachers may have experienced trauma and have their own psychological needs that must be addressed before they can be expected to support their students. Teachers may encounter overcrowded classrooms and be saddled with the task of managing overaged, multilingual, multicultural, mixed-ability classrooms (Winthrop and Kirk 2005). Additionally, Jackie Kirk (2006) observed that teachers are made to perform multiple roles for their students, e.g. the role of a teacher, supporting students’ cognitive and social-emotional growth, and the role of a parent or caregiver, addressing students’ psychosocial needs.

Exploring teacher experiences in the fragile contexts, Jackie Kirk and Rebecca Winthrop’s work with mullah teachers (2007b), female refugee teachers in northern Ethiopia (2007a), and with a teenage girl teacher in rural communities in Afghanistan (Kirk 2008), emerged that people taking up teaching responsibilities in these contexts are often not seasoned professionals. Generally, the most educated person in the community is chosen to teach children. These chosen few may not have completed their own studies and hence are under-confident about their teaching skills. Further, they lack interest in teaching owing to financial compensation either being non-existent, insubstantial, or subject to delays and irregularities. Teachers living in inhospitable living conditions with their own basic needs at risk, facing threats to their own safety and security lack motivation in the teaching profession (Kirk 2008).

Through a teacher stress measurement tool tailored for use in fragile settings, it emerged that teacher stress is one of the major determinants of job performance and hence student learning outcomes (Noah-Pinheiro 2017). By mapping the research conducted in this area with the available inventories, Noah-Pinheiro has coined several factors influencing teacher stress and performance in fragile contexts. Some of them entail organisational characteristics, such as administrative bureaucracy, career advancement, class size, collegiality, degree of autonomy in decision-making, income, job demands, workload, resources, reward and recognition, role conflict and/or ambiguity, and student discipline; environmental factors related to the nature of the teaching job, perceptions or experiences of stress; internal factors pertaining to personality and temperament, attitudes, values, needs, and self-concept, and coping mechanisms adopted.

In a landscape review on teacher occupational well-being in low-resource, crisis, and conflict-affected settings conducted by Danielle Falk, Emily Varni, Julia Finder Johna and Paul Frisoli (2019), a tapestry of individual, school, community, national, and global factors were made explicit. They identified that teacher occupational well-being is highly context-sensitive with subjective well-being indicators varying considerably across countries and socio-demographic characteristics.

## The questions that drove our study

The overarching question examined in this study is—“What belief systems are held by science teachers situated in the conflict-affected zones of Jammu and Kashmir”? In addition, four sub-questions were explored:

- How do teachers believe conflict affects teaching in science classrooms?
- What role do teachers believe that science education can play in ameliorating conflict in society?
- What are the unique needs and considerations for teachers in these areas?
- What unique roles are played by teachers working in these areas?

## What we did to collect data and how

This study utilises qualitative research traditions. Robert K. Yin (1994) observes that this research method is appropriate to answer research questions designed to disentangle complex influences on outcomes. The data were collected during the COVID pandemic when the world was facing travel restrictions. Therefore, online platform of Qualtrics XM was used for the administration of questionnaires and WebEx platform was used for conducting focussed group discussions.

*Questionnaire.* A questionnaire was developed to assess science teacher beliefs about teaching science in the schools located in the conflict-affected zones of J and K. The questionnaire was divided into two parts. Part one consisted of a set of eight questions that sought demographic information from the participants. Part two consisted of ten open-ended questions aimed at retrieving participants’ beliefs about the state of science teaching in conflict zones, ways in which they interact with their students on the issue of extremism and terrorism, how do they conduct science activities and if science textbooks or any other teaching–learning activity gets affected due to conflict in the state.

This questionnaire was developed by both authors. It was sent to an expert working in an education organisation in the state for the last twenty-five years. He validated the questionnaire and guided us to reorganise a few questions. Taking his ideas into consideration, we reworked the questionnaire and administered it to a set of 28 teachers spread across J and K. The purpose of the study was mentioned at the top of the questionnaire, researchers’ identities were revealed on the questionnaire, and it was ensured that participants’ identity will be kept confidential. All the participants were requested to sign their filled-in questionnaires indicating their informed consent to participate in the study. Only signed questionnaires submitted by the teachers were used as the data source.

Only 19 teachers submitted signed responses to the questionnaire. Out of the nineteen, eight were from Jammu city, one from the urban city of Muzaffarabad located in Pakistan administered Kashmir (POK), and the other ten were from four different schools in Kashmir that included eight responses from Srinagar city, and two responses from a rural village in Budgam.

**Fig. 2:**
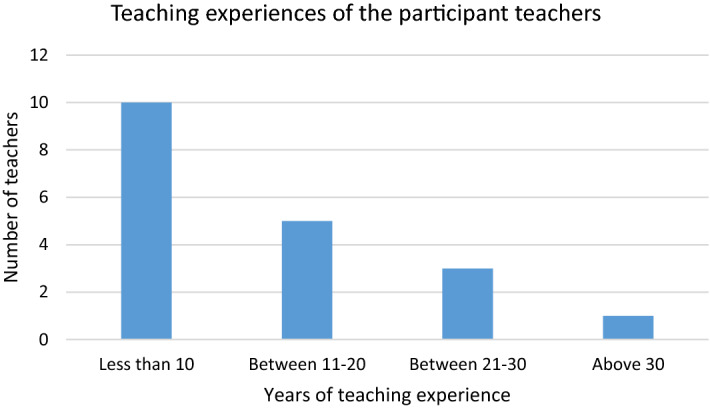
Teaching experiences of the participant teachers

Three teachers who responded to the questionnaire were teaching elementary grades while all the others had teaching experience with either secondary or senior secondary grades. The average teaching experience of the respondents was 11 years. Further details are included in Fig. 2.

*Focused group discussions.* We sent out a request for a focussed group discussion (FGD, henceforth) to those teachers who responded to the questionnaire. Only 9 out of 19 teachers agreed to our request. Keeping British Educational Research Association (BERA) ethical guidelines into consideration (BERA 2011), we revealed our identities, declared the purpose of the study, and assured that participant identity would be kept anonymous during the entire research process and publication of research findings. The first FGD was conducted with a set of five teachers from Jammu (four females and one male). The second one was with four teachers from the Kathua and Rajouri regions of Jammu. It was interesting to note that none of the teachers switched on their cameras during the online interaction in FGD2 which was not the case in FGD1. Despite repeated reminders, no teacher either from Kashmir or from POK agreed to a focussed group discussion. Both the authors speculate that teachers from Kashmir and POK did not participate for the fear of being identified as education practices in J and K is a highly politicised issue.

During this study, we did not meet with any of the participant teachers physically due to COVID-19 restrictions and safety precautions. FGDs were our first virtual-mode-face-to-face interaction with them. In order to motivate them to respond to our questions, we used storytelling as a potential technique. Teachers were encouraged to respond to imaginary incidents/stories that were true to their reality.

Multilateral ways of thinking emerged from FGDs. Teachers preferred to provide brief responses in the beginning. However, with stories in place, they opened up their realm of experiences and provided anecdotal evidence from their personal or professional lives. To illustrate, on the issue of the Hindu and Muslim relationships in the state, all the teachers in both the FGDs mentioned that both Hindu and Muslim communities intermingle in their personal spheres of life. They generally celebrate each other’s festivals. To illustrate, a teacher in FGD2 observed:

Jab meri shaadi hui thi, my in-laws belonged to one of the border areas, Rajouri. I was worried… kahin terrorist to nahin honge...Rajouri to apko pata hi hai ki

border area hai (*When I got married to a man living in Rajouri which is a village lying on the border, I was sceptical of my in-laws... I wondered can they be terrorists…*)... (laughed)... We have only two Hindu families and all others are Muslims in our community. When my mother-in-law was ill, main akeli thi … saare padosi aa gaye the madad ko (*I was alone and all my neighbours came for my support*) (FGD2)

Contrary to this, one teacher observed that it may happen that one of your close friends belonging to a particular religious community falls prey to anti-social elements operative in J and K. You only come to know of their activities through newspapers when they are caught causing conflict. People feel betrayed during such occasions and tend to develop a negative attitude towards specific communities.

Apart from these teachers, we also included one Kashmiri teacher narrative that were recently published in Pari education to draw a holistic picture of the teacher beliefs in J and K (https://pari.education/articles/in-kashmir-we-cant-fulfill-our-role-as-teachers/).

During FGDs, teachers interacted as a group and in most cases, an alignment was observed in their views. For example, in FGD 1 all the teachers promoted an egalitarian view of education. They observed that ostracising any religion due to the unlawful activities of a few people is not justified. All of them reiterated that they consider all children equal and are committed to their student well-being.

## Making sense of the data

Thematic analysis is a qualitative research method that can be widely used across a range of epistemologies and research questions. Virginia Braun and Victoria Clarke (2006) observed that this method is useful for identifying, analysing, organising, describing, and reporting themes found within a data set. All data generated were transcribed and read iteratively to locate concepts being represented by the data. Both the researchers independently analysed the data and only themes that were figured in both researchers’ analyses or were accepted by both for inclusion are presented.

Open coding procedures as delineated by Juliet Corbin and Anselm Strauss (2008) were utilised which involved continually asking the following questions:

- Which category does this incident/ word/ phrase allude to? For example, teachers’ description of frequent school closures owing to extremist activities in J and K refer to effect of conflict on teaching; teacher quotes, such as, ‘after my husband’s transfer to J and K, I joined as a teacher in a school in Jammu… *pehle dar lagta tha phir samajhne lagi* (Though I was scared to teach in Jammu schools in the beginning I later understood the situation)’ allude to a biased view held by the teacher towards specific religious communities.
- What are the similarities or differences between the two emerging concepts? And, so on. For example, on certain occasions, teachers observed that they hold egalitarian views towards all sects of the society, however, deeper probing suggested that they held an ‘otherness’ view towards a few segments of the society. These instances often led to the emergence of two contradictory themes; for instance, one thread points towards the teacher’s role as an equaliser, while the other thread refers to a biased view. To counteract these differences, both the researchers revisited the dataset iteratively to identify the dominant themes coming out from the majority of teacher voices. And reasons for exceptional voices not aligning with the major theme were probed. In cases, where avenues for further data collection were not possible, we have reported the conflicting views to describe a holistic picture of the field.

Words/themes/and other data pieces alluding to a particular theme were colour-coded. Processes of bundling, grouping similar units, and deletion of synonymous units were utilised to arrive at final categories as delineated in the research findings.

Both authors independently conducted the thematic analysis to arrive at the data themes. The independently generated themes by the two authors revealed a high degree of agreement. The data pieces were revisited collaboratively to discuss the disagreements and to develop a consensus on the themes.

One anonymous focussed group discussion transcripts and questionnaire responses were shared with a science educator working in J and K. He is the same person who had supported us in getting access to the participant teachers in the study and had validated our questionnaire. He was requested to identify themes emerging from the data. Using the WebEx platform for online discussion, disagreements were addressed to finally arrive at the themes used to discuss the research findings of the study.

## What we learned

This section presents science teacher beliefs held by the teachers in the conflict-affected zones of Jammu and Kashmir. Collected data using a combination of surveys and focus groups with nineteen teachers who had a wide range of teaching experience in the region, revealed several themes pertaining to science teacher beliefs. The learnings learned from this study are discussed under the following themes: Teacher beliefs on how conflict affects classroom practices, conflict and teaching issues, multifaceted role of teachers in conflict-affected zones, the role of science education in ameliorating conflict, and changing role of teachers during three decades of active conflict in J and K is reported. The following abbreviations are used to discuss the findings: QRn: Questionnaire response with ‘n’ representing the number of the participant teacher, FGD1: Focussed group discussion 1; FGD2: Focussed group discussion 2.

## Teacher beliefs on how conflict affects classroom practices

Situated in the context of conflict, it was crucial for us to understand how teachers believed conflict was affecting their classroom teaching. Except for two participants, all the other respondents to the questionnaire reported a negative impact of conflict on their teaching. They believed that conflict in the region is not congenial for learning. For example, conflict in the region leads to frequent closure of schools which causes disinterest in studies amongst students. Also, students get disturbed and become disinterested in learning influenced by an implicit fear of the school environment. In government schools, many students come from weak educational and financial backgrounds and conflict further compound the educational crisis which these children face. One of the teachers mentioned in her questionnaire responses:

Students do not show readiness to get involved in science activities. They want to leave… they get mentally disturbed during unlawful happenings in their environment… (QR5)

Contradictorily, two of the respondents observed that any act of terrorism does not impact the psychosocial school environment and schools continue to provide students with a safe learning space despite tensions being commonplace in the outside world. One of the teachers, out of these two, teaching in the Jammu region with one year of teaching experience, observed that Jammu is hardly affected by conflicts. While the other teacher with fifteen years of teaching experience in the Jammu region mentioned in his questionnaire responses that despite incidents of violent extremism, classrooms remain safe places for children to continue with academic work. We further probed this teacher during the focussed group discussions where he candidly noted that he was not sure about the confidentiality of participant responses to the questionnaire data. Therefore, he had simply given “safe answers” to avoid any threat from anti-social elements in the state if the responses were revealed. This strongly suggests that many people who live in a constant threatening environment where trusting organisations does not come easy to them. Another teacher in FGD2 added, “comments made by people in our region are exacerbated in news channels”. It is difficult to identify who is a trusted source, therefore, we prefer to keep silent on any issue related to the state.

When asked if teaching in their geographical location was different from teaching in other states of the country, teachers reiterated that teaching in conflict-affected zones is challenging as they have to deal with students who face trauma, uncertainty, and insecurity in their personal spaces. Some other issues raised by them in the questionnaire responses are as follows:

I think teaching in non-conflict areas is more effective as the interaction between teachers and students is more as compared to conflict-ridden areas (QR3)

In conflict-ridden areas, students are disturbed and face many difficulties and cannot think about what is good for their future. Internet facility is at maximum times down, ... students get frustrated with not getting the benefit of online education (QR2).

In conflict zones, there is always uncertainty. You never know what is going to happen next (QR11)

Teaching is totally different in conflict-ridden areas severely affecting teaching and learning. In lesser disturbed areas, generally speaking, students are calmer, more affectionate, and disciplined but it is vice versa in conflict-ridden areas. Teachers are not able to give lectures with full enthusiasm because students are not classroom-ready… they are occupied with other stuff in their immediate environment… you know… militancy, bomb attacks, deaths… (QR17)

It is very tough to teach in this situation. Students, as well as teachers, remain in dilemma and uncertainty (QR9)

The environment plays an important role in learning. The free and fair conditions make a learner more cooperative and lets them come up with excellent self-expressions. On the contrary, conflicts shrink the cognitive capacity of learners (QR8).

Teaching in Kashmir is a challenge and more so in the last few years. We suffer ongoing curfews, and closure of educational institutes and other workplaces. I sometimes feel I shouldn’t have become a teacher because we can’t fulfil our roles as teachers in Kashmir (https://pari.education/articles/in-kashmir-we-cant-fulfill-our-role-as-teachers/).

Clearly, teachers in conflict-affected zones are charged with the responsibility of teaching children in poor mental health conditions and facing psychosocial stress. Additionally, teachers reported that conflict affects classroom teaching in other multiple ways. It may be a direct influence on the school calendar, paucity of resources, or an indirect implication of lack of teacher professional development on teaching–learning processes in the classrooms.

## Conflict and professional teaching issues

### Teacher professional development

Teachers noted that teaching is a continuous process of learning. They need to update themselves to better adjust to the changing educational landscapes. They reported that lack of professional training of teachers in the state is a norm. As an example, during the COVID pandemic, teachers yearned for hand holding for the advancement of technological pedagogical skills to enable them to conduct meaningful online learning sessions for their students. Some of the teachers’ comments were as follows:

The world is advancing with technology. It is the responsibility of a teacher to stay updated in order to prepare students for all the upcoming changes in society and the world. The classroom is a mini-society itself, but due to a lack of professional development, I am not able to… help my students… to grow… (FG1)

They (referring to teachers) should work night and day to boost their knowledge (QR8).

### Availability of resources

Teachers urged for better-equipped classrooms having adequate teaching–learning resources. In the questionnaire, teachers demonstrated a need for resources like computer facilities to teach science, well-equipped laboratory space maintained in proper condition, and attractive classrooms spaces having science posters etc.

While probing further during the focussed group discussion*s,* teachers observed that the damage caused to the schools in the decade 1990–2000 was huge. They observed that getting school facilities on par with facilities for science teaching in similar schools in the rest of the country is a challenge. In addition to this, they indicated that in recent times funds dedicated to improving schools were perhaps moving towards conflict-resolution in the state, thus compromising the educational quality in the schools. Furthermore, restrictions to movement in the state owing to sporadic attacks, militancy, and other travel restrictions impacted the way science was taught in the classrooms. Insights into prevailing situations can be visualised from the teachers’ comment in the questionnaire:

Science activities are influenced as no science trips get arranged at proper times (QR12)

Teaching aids are not available for demonstration purposes (QR6)

More smart classes are required… (QR2)

### Representation of conflict in science textbooks

One of the questions posed to teachers in the questionnaire inquired about the effect of conflict on textbooks. To this most of the teachers responded that science textbooks hardly get affected. However, textbooks are not updated on a regular basis to keep up with the scientific advancements in the world. Only one teacher, teaching middle grade science, noted the following:

Yes, various new issues and conflicts are added in textbooks every year, so that children prepare themselves for upcoming challenges (QR8).

She did not participate in the focused group discussion; thus we were not able to elicit more information from her. Marie Lall (2008) analysed how in India the BJP-led government (1998–2004) and in Pakistan, the government under General Zia-ul-Haq (1977–1988) rewrote the curricula and changed textbook content to create the ‘other’ in order to suit their respective ideologies. Drawing on the original textbooks and teacher interviews in both countries, she concluded that fundamentalisation found within textbooks may lead to the development of antagonistic national identities among children in both countries opposing each other’s definition of history and self.

### School calendar

Teachers reported that the school calendar gets severely affected due to terrorist activities in the state. This irregularity of the school calendar consequently impacts the teaching–learning processes in the classrooms. Some of the questionnaire responses shedding light on this issue are as follows:

It first happened after the abrogation of Article 370 on August 5, 2019. Schools shut; we were put under a communication blackout to prevent riots. The internet was blocked and so students couldn’t download study materials. Online education was not an option (https://pari.education/articles/in-kashmir-we-cant-fulfill-our-role-as-teachers/).

No sooner than there is a militant-related incident in the area; schools are the first institutions that get closed, impacting students and teachers (QR1).

As there are a fewer number of working days so interactions are less that affects the day-to-day teaching-learning process (QR13).

Attendance level is low and whenever there is disturbance children don’t come… (QR10)

Most of the students on the days of conflict rarely come to school. They even lack concentration and seem to be mentally disturbed… (QR14)

Teachers mentioned that discontinuity in school attendance results in non-rhythmic attendance in schools which creates a disconnect not only in studies but also in teacher-student relationships.

### Multifaceted teacher roles

Research conducted in fragile contexts has noted that teachers are often tasked with the job of playing several diverse roles while teaching in these areas (Kirk and Winthrop 2013). Similar observations were arrived at through this study. 

Teachers recognised the following *qualities of a competent teacher* in conflict-affected zones:

A good teacher can change the whole world by inculcating the good human traits among learners, besides empowering them with skills of serving the people and making life happiest (QR6)

Be honest always as far as teaching is concerned

Be strong, Be original, Be innovative, Be Collaborative, Be dedicated (QR8)

Teaching is a noble profession… work hard… be friendly with students (QR1)

The teachers were cautious to *portray their best character or behaviour* in front of their students as they believed that students mirrored behaviours exhibited by them. For example, one teacher stated:

It’s necessary for a teacher to be honest with his/her job role and responsibility. Students always follow what the teacher does and reflect on them. Teachers should be careful about what he/she does and also make sure of fulfilling the commitments he/she makes. Once a student finds the teacher not sticking to his/her words then the student shall also start ignoring the teacher (FGD1).

An overarching theme coming from both sources of data was that all the teachers recognised themselves as *humanists* responsible for passing moral values to their students. This observation is in line with UNICEF’s study (2005) where it emerged that during the conflict, teachers’ roles—as counsellors and mentors—magnify in importance. All teachers recognised that they have to be impartial and treat all their students equally despite them belonging to different castes, creeds, religions, and gender. This was further elaborated by their comments on the *universal human value of equality.* To illustrate this finding:

It does not matter from which religious group our students come from, when they come to us they are our students and we treat them equally (FGD1).

Children need to know ki ek hi religion ke log terrorist activity mein involve nahin hote. (Children need to know that people from any particular religion don’t get involved in the terrorist activity)… for example, in kissan andolan in Dilli (a farmer sponsored agitation in Delhi)… people from all the religions are participating which is also leading to violence… (FGD2)

We celebrate all the Indian festivals in schools whether it is Id, Diwali, Gurupurab… (FGD2)

However, further probing during focussed group discussions revealed that teachers held deeply entrenched biased views about cultural and religious sects of the society. They conceded that over the past few decades, the representation of Muslim students in mainstream schools has increased. This is driven by Muslim families from small villages investing a lot of time, money, and effort to send their children to mainstream schools in cities. Perhaps Muslim parents are aware of, what some of the teacher participants believe, that *madarsa education* which is provided to Muslim children is non-progressive and needs to be replaced with a form of science education that is instrumental in developing a rational outlook in the community. While probing further teachers’ views on rational outlook, they observed.

Our children should understand that no religion promotes antagonistic views against human civilisation… unhe sahi galat mein differentiate karna aana chahiye (Our children *should be able to differentiate between right and wrong*)

Teachers noticed that science education empowers people to differentiate between fact and fiction.

Teachers’ responses to questionnaire alluded that there has been a major change in the choice of careers chosen by the local diaspora which included both Hindu and Muslim communities residing in the region.

Very few Kashmiri’s in the past would aspire to take up administrative roles in the Government of India, which with the passage of time has changed… (QR4).

In addition, teachers observed that they have to be very cautious in the types of examples they choose while teaching science. They need to ensure that any content they teach should not have any religious overtones that may disturb religious sentiments of the children. For example, a teacher observed that while discussing the medicinal value of plants she used to give the example of a tulsi plant (basil) which is revered in Hindu religion. A teacher suggested that using this as an example may offend other religious communities. In her words:

Tulsi ko cough cold ko treat karne ke liye sadiyon se use karte hain (*Tulsi plant is used for the treatment of cough and cold since ages*)… kuch bache sochte hain tulsi hi kyon koi aur example kyon nahin (*some children think that why am I only explaining using the example of tulsi plant why not any other plant?*)… when I realised that some children are getting offended… I started being careful… bahut saavdhan (*very careful*)… (FGD1)

A few teachers indicated that irrespective of religion, women’s education rates are significantly low in J and K. Low numbers of women who have gone to school and finished at least the primary grades have repercussions on their children's education. Such mothers may not be able to support their children in school-related academic tasks as they themselves lack that academic training provided in schools.

Overwhelmingly, teachers have assumed the role of counsellors for their students. They believed that being a teacher it is their responsibility to lead their students in the right direction. They strive to provide a healthy and healing classroom environment where children are free to share concerns with trusted adults and peers. To illustrate:

Students aren’t the only ones accountable to exhibit respect. Teachers also need to have respect and responsibility towards students especially under current difficult situations. I feel the responsibility towards my students (FGD 2)

To involve students in the teaching-learning processes in conflict zones is too difficult. Because you have to be an extraordinary teacher to involve a mindset that is under stress (FGD1).

I tell my students don’t pay any attention to what is happening outside, to focus on their studies, and prepare themselves for the competitive exams (QR13).

Additionally, teachers realised that one of their major roles is to provide children with *cognitive protection* in the form of new information, skills, and attitudes which can foster appropriate problem-solving, decision-making, and conflict-resolution amongst the children and young people.

There have been occasions when such things happened around our school. I asked my students to concentrate on their future life and not involve themselves blindly, otherwise you may invite trouble not only for yourself but also for your parents (QR3).

Yes, once while teaching my students in a classroom some conflict occurred outside our school premises. At that time I did not pay any heed towards that incident and kept on going with my work and students too did the same when they found me showing no response to that conflict… After finishing my work I advise my students never to indulge themselves in such conflicts where they will have to repent for life long (FGD 1).

We organise dramas in the morning assembly. Society mein jo evil hain… every Thursday and Saturday… I am the activity incharge. Last drama mera Dilli tak gaya tha… It was totally based on Muslims and non-Muslims… army day mein bhi select hua tha...do ladkiyan dost hain...ek Muslim ki… ek hai Hindu… bachpan se saath rahein hain… hum apne dresses bhi exchange karte hain… we guide our children during assemblies… (FGD 2)

(*We organise morning assemblies around topics of conflict flashing in the newspapers on every Thursday and Saturday. I am the activity incharge. Last drama created by me was very well appreciated. It was selected for the army day and even shown in the Delhi branch of our school. The plot of drama included two girls, one belonging to a Hindu family while the other belonged to a Muslim family. These girls grew up to become a doctor and a pilot respectively. They had been friends since childhood and they used to exchange dresses*… *this is how we guide our children during morning assemblies*)

I organise group discussions in my classrooms… we promote cordial relationships among all children belonging to different religions… We protect our students to develop antagonistic views against any particular religion (FGD 2).

Also, teachers saw a sense of urgency in *delivering their curricular goals*. They believed that inaction or delays in teaching owing to conflict in society often gets counterproductive to students’ academic growth. They suggested:

Students come to school despite so many issues in their lives… they spend their valuable time expecting to learn something meaningful and important in school. If I spend most of my class time discussing these disturbances in the region, their time gets wasted which reflects poorly on their results (QR14).

The more we linger, the more we shall find ourselves with little time… We struggle in meeting academic as well as non-academic goals set for our students (FGD 1).

Some students could be weak and might not be able to catch up with the pace. It’s my responsibility to always stick with the possibility of students progressing in their academics and in their life as well. All students don’t have the same capacity for learning. They have different learning styles so I should exercise different ways of explaining core concepts (FGD1).

## Teacher belief on the role of science in ameliorating conflict

Most teachers realised the role of science in the reconstruction of stable societies. They believed that it is important to develop a questioning attitude and critical thinking skills among children. This will prevent them from getting brainwashed by anti-social elements operatives in the state. Teachers observed:

I encourage open dialogues in my science classrooms. You know… science encourages asking ‘why’ for everything they see around them… I tell you… this questioning attitude will help them in making life choices… (FGD1)

Scientific method in itself is an eye opener… not accepting things, any religious dogma, any voice…science is an attitude in itself… in fact… science is a religion… (FGD1)

As science is a vast subject, I try to engage more and more students so that they may not get involved in any kind of unrest-related activities (QR3)

By developing interest among students so that they don’t deviate from their path Science helps to develop a rational approach (QR9)

Science students are highly submissive and obedient, they can be easily moulded in the right direction as most of them have strong affirmation regarding their goals which in turn is related to the nation’s progress (QR14).

Teachers reflected upon the changing trend in students’ choice of discipline in senior secondary grades. They observed during focussed group discussions that many students in their classes are opting for humanities and commerce streams instead of scoring well in secondary grade science examinations. They concluded that this choice is made due to certain prerequisites, e.g. completion of a specified number of science practicals which is impossible in conflict-affected zones as they cannot come to school regularly for practicals*.* However, teachers seem to be committed to reigniting students’ interest in science. They believed that studying science will not only awaken children’s critical mental faculties but will help them rise on the economic ladder and pave the way for a peaceful tomorrow. These changing trends are making teachers take a *leadership* role in society, thereby leading students towards roles valued by society.

Science pays the way forward for economic upliftment which helps in mitigating political and social unrest (QR13)

The teaching of science will produce a good human resource that will work for the betterment of life on earth, where there will be no place for conflicts (QR15)

Peepal ke ped ke chakar lagana...paranormal activities… herbal products ko use karna… meri mummy ko sugar thi… unhone neem, methi dana le lekar apni body damage kar li… I am a complete non-believer of these things… isliye, science ka role society mein bahut zaroori hai… (FGD 2)

(*Teacher narrated commonly held superstitious beliefs rampant in the society, such as taking rounds around the fig tree, believing in paranormal activities, and taking herbal plants as medicines for cure of diabetes. She suggested that her mother used to take neem plant and fenugreek seeds as herbs to cure diabetes. According to her, it damaged her mother’s body. She said that she is a non-believer of superstitions and herbal products. Therefore, the role of science is crucial in society*)

Teachers, both in questionnaires as well as focussed group discussions, reiterated that they believed in the importance of activity-based science teaching wherein activities are situated through real-life authentic experiences. In addition, they preferred diagram drawings, group discussions, and biological identifications through specimens, charts, and slides, as examples. This indicates to us that teachers were motivated to teach science in such a manner that students could relate it to their everyday lives.

Student-oriented learning is my preference, usually, I bring many specimens to the classroom from lab items available. I try my level best to use them to convey ideas (QR10).

## Changing role of teachers during three decades of active conflict in Jammu and Kashmir

Teachers in our dataset belonged to diverse time zones with varying years of experience in the classroom. Some had as many as 31 years of teaching experience while others were relatively novices having one year of teaching experience (see Fig. 2). However, all of them witnessed disturbances in their educational journeys. This had made them aware of the difficulties faced by students, thus they were *cognizant of student needs* in conflict-affected regions. Situated in the context of a territory affected by conflict for the last three decades, it seemed important to inquire if they have witnessed any changes in their teaching roles or educational journeys.

Teachers observed that during the pre-militancy period (1980–1990), science education entailed didactic methodology with a strong emphasis on curriculum coverage. They observed that during that period curriculum was comprehensive and science experimentation was scarce. In the following decade (1990–2000) when militancy activities peaked, science teaching suffered badly due to frequent school closures which affected school attendance. Also, schools were badly resourced, which led them to recourse to didactic ways of teaching and learning science.

Following the period of active conflict, the last two decades (2001–2019) could be seen as settling down period for the region. People have started accepting sporadic terror activities and a heavy presence of the military as a new norm for the region. In the twenty-first century, people in the region are seemingly more focused on their career aspirations. Teachers observed that science teaching has improved considerably during this period. They used innovative pedagogy, technology, and laboratory work to ensure those science concepts are fully developed amongst the student populace.

While examining their views on teaching science during the COVID pandemic, they observed that though they have eventually developed the knack for online teaching, nevertheless, frequent internet disruptions pose a problem.

Teachers in FGD 2 observed:

I can recall that once when I was in college… there was turbulence… kuch land se related tha… amarnath se shayad… mera college teen mahine tak band raha tha… I wish aaj ke jaise technology hoti… we are still teaching our students when the schools are shut down… I am hopeful jaise ab situation sudhar rahi hai...next generation may not face disruptions in their schooling in future…

(*There was a period of active disruption in the valley... some issue related to land... perhaps related to amarnath...my college remained shut for three months... I wish we had technology access at that time the way we have it nowadays. We are teaching our students even when the schools are shut using technology. I am hopeful that with technological innovations coming generations will not face discontinuity in schools even in circumstances of schools being shut*)

Another teacher in FGD2 observed:

With the new education policy (referring to New Education Policy 2020), things have become challenging… mere time mein bahut saara syllabus padhte the... ab to small packets information hi dene hain… bachon ko burden nahin dena… har period ka time ab sirf 30 minutes ka ho gaya...jab tak seedi chad ke class tak pahuncthe hai...topic discuss karke discussion shuru hota hai… tab tak class time over ho jaata hai… bache ab computer jaise bana diye hain… thoda feed karo… thoda hi bahar aayega…

*(New Education Policy (referring to New Education Policy 2020) have added challenges to the educational practices in schools. We used to have a comprehensive syllabus to study during our school days (referring to a time period when she was herself a school student), however, now the emphasis is to reduce curriculum overload on the children. We have to provide them small packets of information. It’s like the input-output system of the computer wherein we are feeding a small amount of information to students and they regurgitate those small packets during examinations. Moreover, now the duration of each class period is reduced to 30 minutes. Till I reach my classroom after climbing so many stairs, I explain the concept and begin discussion...class time gets over).*

It is discernible from the above-mentioned quotes that teachers held mixed feeling towards changes in educational practices. Some of them were looking at the positive side of technology in continuing education during school closures while others seemed unhappy with a few changes brought in with the introduction of the new education policy.

**Table:** 

**Vignette** **Personal reflections of the author** I, Rekha Koul, was born in the then state of Jammu and Kashmir in the decade of sixties of the twentieth century and grew up and have fond memories of my schooling in the seventies. My father was a senior administrator working for the Department of Agriculture in the state. His nature of job took him all over the state and as a young girl I moved with him and other family members. For my early years of schooling, I went to three different schools in Doda, Jammu and Srinagar (see Fig. 1). After moving to Srinagar in my teenage years, I along with my family continued to live there until I could not go back to Srinagar my HOME when I had gone to Delhi for a short vacation in January 1990 My school days in Srinagar were generally very peaceful but would witness occasional violent demonstrations by the locals, which to a logical mind seems senseless. Reasons behind these demonstrations could be like prophet Mohamad’s picture being published in a book in some parts of the world, Pakistan losing a cricket match with India or the hanging of Mr Zulfikar Ali Butoo the Ex-Prime minister of Pakistan. Minority community (Hindus) would be targeted in the violent episodes though they had no role in these events. The majority group saw minorities as agents of India living in Kashmir and I belonged to the minority community because I was born in a Hindu family. On the other hand, both communities co-existed. The majority of my friends belonged to the Muslim community, which was normal. My school was run by a Hindu religious sect and we had many Muslim students in our school who would participate in Hindu rituals as part of our moral science classes There was a marked difference in behaviours in the second half of the decade of the 80’s. Targeted killings especially of noted personalities from minority groups, and bomb blasts in crowded places started which created fear in the minds of the Hindu diaspora living in Kashmir. These events also impacted the functionality of educational institutions. On the night of 19^th^ January, 1990 a general call through loudspeakers was made for all non-Muslims to leave Kashmir, resulting in half a million Hindus leaving their homes for Jammu where the majority community was of Hindus or the rest of India or anywhere else in the world By 1990, I was mother of a 3-year-old child. I had left home for a short vacation but had nowhere to go. I could not access my money saved in the banks of Srinagar and accessing it from other parts of the country was not possible as banks were not computerised then. The situation of other members of my extended family was no different, thus there was no one to whom I could turn for help. Both my partner and I were lucky to find work in areas of our specialisation in Delhi, thus continuing our lives. I was lucky enough to given an opportunity to work at the prestigious Lady Irwin College of the University of Delhi After a few months we did join our extended family in Jammu, where they had made make-shift arrangements for us. Eventually, Jammu became our home, but life never seemed normal. Life during this time was full of struggles. These struggles were multi-fold. Firstly, I had to come to terms that I can’t go back to my actual home and alternative provisions need creating, which was emotionally as well as mentally taxing. Most nights were sleepless, and days were spent discussing the plight we were going through. Despite having a regular reasonably well-paying job financially, I was doing tough. Setting up a new home from scratch was draining me out monetarily. Some assistance was provided to unemployed people from the Government but since I was employed, I didn’t qualify for any. In addition to emotional and economic strain, I had to make pace with the culture of the new place. I had moved from a cold climate to a hot climate, which came with its own challenges, e.g. how to dress, what to eat, how to keep your home cool etc. In addition, I experienced violent activities even in Jammu during the decade of 90’s. This uncertainty lead us (my family and I) to immigrate to Australia, which is home now I continue visiting Jammu and Kashmir over the years. Visiting my extended family was logical to keep in touch with my Kashmiri roots. This was further reinforced by making annual weeklong visits to Srinagar between the years of 2012 to 2018. I have not been able to go to Srinagar after the revocation of Article 370, as it was not thought to be safe for me and haven’t visited Jammu since January 2020, travel restrictions imposed due to COVID-19 Kashmiri’s over the years had realised that violence is not helping them in moving forward and the militant activities had greatly reduced. In August 2019, the special status given to the state was repealed which resulted in a few months of curfew followed by the Global Pandemic COVID-19. I hope to post COVID-19, Kashmir sees a return of normalcy and Kashmiris get to living in peaceful atmosphere and children experience regular schooling	

## What we wish to share with you

Teacher beliefs influence the ways they orchestrate their classroom teaching, and how they interact with students, parents, and the school community (Bybee 1993). Though teacher beliefs have been widely explored by education researchers there exists a paucity of research studies examining science teacher beliefs working in conflict-affected areas, especially in south-east Asia. Acknowledging this gap, this study examined science teacher beliefs working in conflict-affected regions along the border of Jammu and Kashmir.

This study revealed that teacher beliefs are also borderlands and are complex and not unidimensional constructs; rather they are an outcome of the complex entanglement of social, emotional, psychological, historical, and educational journeys of the teachers. A rich narrative of teachers’ personal growth trajectories intertwined with contextual factors and their concomitant impact on teacher beliefs can be noted in the excerpts we shared from the teachers. We learned that teachers in the study unanimously believed that conflict negatively affects classroom teaching. They highlighted specific ways in which conflict influences classroom teaching. Some of them entail frequent school closures, dearth of high-quality teaching–learning resources, an absence of well-resourced laboratories, and a lack of teacher professional development.

Despite challenges posed by conflict in science classroom teaching, it was clear that teachers were committed to providing a healthy and healing classroom environment for their students. They observed and related to us that those teachers in the conflict-affected zones are tasked to play multiple roles of a counsellor, mentor, and humanist along with the usual teaching responsibilities of curriculum coverage. Also, even though conflict occurs outside the school premises, its reverberations can be felt inside the classrooms in the form of psychosocial distress, fear, and distrust among students and teachers. To try and counter psychosocial distress, they employed a variety of pedagogical approaches that dealt with conflict-resolution in schools.

All teacher participants of this study believed that science education has the capacity to develop a rational outlook and critical thinking among students. They considered it to be a channel through which the decision-making capacity of students can be sharpened, thus preventing them from falling prey to anti-social elements active in their areas.

However, their narratives evinced deeply held implicit beliefs of religious biases. It was evident that teachers themselves were unaware of their biased views which they have either carried forward from their childhood experiences, other personal experiences, or from professional experiences developed from teaching in various parts of the world. They noted that though they propagate egalitarian views as professionals, they prefer to remain careful while teaching multi-religious groups present in their classrooms. Strategic distancing from members of a particular religion emerged on a few occasions. For instance, some teachers observed that using specific examples of herbal plants that are revered by a particular religion may offend children belonging to other religions. This is an interesting finding. Examples of herbal plants could be considered by some to be leveraging students’ funds of knowledge. The complexity of the classroom makes it difficult to enact funds of knowledge framework. I think it would be valuable to explore this as a challenge in teaching science in J and K.

Clear distinctions of changes in teacher beliefs over the last three decades of active conflict in the region unfolded. Some of them were hopeful that technological advancements would facilitate continuity in schools even in conditions of school closures due to conflict in the region. While others reflected on certain managerial issues that have emerged due to changes in education policies over the period of time. In all, teachers demonstrated mixed feelings towards changing socio-cultural-political-educational landscape in J and K.

This study provides a vivid picture of science teacher beliefs in conflict-affected zones of J and K. It is suggested that teachers need to be supported in examining their own implicit beliefs about various issues latently present in the conflict zones before they are charged with the responsibility of supporting students and the community at large. Having said this, it should be noted that this study was carried out with a small sample of teachers. The data set had a skewed teacher representation from different parts of J and K. No teacher either from Kashmir or Pakistan governed Kashmir participated in the focussed group discussions. Also, teachers from only one religion and only one male teacher participated in both the focussed group discussions, thus limiting the scope of findings to illuminate a real picture of the issues under consideration. Additionally, this study was carried out during COVID pandemic when owing to travel restrictions authors used online modes of data collection. This mode of interaction has its own limitations in terms of inability to capture participant gestures, colloquialisms, and facial expressions (as cameras were turned off in one of the FGDs). Given the aforementioned limitations, this study does offer some windows into the pedagogical issues that teachers in conflict zones must contend with.
